# Localizing Age-Related Changes in Brain Structure Using Voxel-Based Morphometry

**DOI:** 10.1155/2017/6303512

**Published:** 2017-01-17

**Authors:** Shu Hua Mu, Min Xu, Jun Xiu Duan, Jian Zhang, Li Hai Tan

**Affiliations:** ^1^College of Psychology and Sociology, Shenzhen University, Shenzhen 518060, China; ^2^Neuroimaging Laboratory, School of Biomedical Engineering, Shenzhen University Health Science Center, Shenzhen 518060, China; ^3^Center for Language and Brain, Shenzhen Institute of Neuroscience, Shenzhen 518057, China; ^4^Shenzhen University Health Science Center, Shenzhen, China

## Abstract

*Aim*. We report the dynamic anatomical sequence of human cortical gray matter development from late childhood to young adults using VBM and ROI-based methods.* Method*. The structural MRI of 91 normal individuals ranging in age from 6 to 26 years was obtained and the GMV for each region was measured.* Results*. Our results showed that the earliest loss of GMV occurred in left olfactory, right precuneus, caudate, left putamen, pallidum, and left middle temporal gyrus. In addition, the trajectory of maturational and aging showed a linear decline in GMV on both cortical lobes and subcortical regions. The most loss of gray matter was observed in the parietal lobe and basal ganglia, whereas the less loss occurred in the temporal lobe and hippocampus, especially in the left middle temporal pole, which showed no decline until 26 years old. Moreover, the volumes of GM, WM, and CSF were also assessed for linear age effects, showing a significant linear decline in GM with age and a significant linear increase in both WM and CSF with age.* Interpretation*. Overall, our findings lend support to previous findings of the normal brain development of regional cortex, and they may help in understanding of neurodevelopmental disorders.

## 1. Introduction

Detailed evaluations of human brains can be performed in vivo in a safe and noninvasive manner, even in healthy developing pediatric patients, due to recent advancements in magnetic resonance imaging (MRI) [1–3]. Early pediatric studies using MRI found that in normally developing children the amount of white matter increased in a linear manner as the patient aged, while the gray matter started decreasing after 6-7 years of age and continued through adolescence [4]. Specifically, the gray matter first decreased starting between 4 and 8 years of age in the primary sensorimotor and dorsal parietal regions close to the interhemispheric margin. From there, the gray matter loss then spread laterally and caudally to the temporal cortices and anteriorly to the dorsolateral prefrontal regions. Early maturation occurs in the regions that perform the most basic functions, for example, processing of sensory stimuli and movement [3]. Recent studies have used a number of methods to characterize regional gray matter changes over the course of a person's life. Gogtay et al. (2004) studied developmental anatomical changes to human cortical gray matter in those aged 4 to 21 years, focusing on changes that occurred in 17 regions of the cortical surface [3]. In a similar manner, Sowell et al. (2003) measured the density of gray matter in 176 healthy people aged 7–87 years by mapping 35 sulcal and gyral landmarks for each hemisphere on both the lateral and interhemispheric surfaces [5]. However, previously published studies have reported on only a minority of regions of the brain and changes in the gray matter in a number of regions, for example, the subcortical regions, have yet to be studied using the approaches detailed above.

An unbiased technique for assessing the whole brain is voxel-based morphometry (VBM), which can be used to measure the cerebral volume of regions of the brain and concentration differences between tissues in structural MRI. This method evaluates differences in gray and white matter density voxel by voxel using spatially normalized and smoothed gray or white matter maps [6, 7]. VBM has been utilized in several studies to investigate the relationship between changes in gray matter and age [8] and has been confirmed as an accurate technique using ROI and functional analyses [9, 10]. Furthermore, analyses can be strengthened by using ROI in conjunction with VBM, where cerebral regions can first be defined with VBM analyses and then studied in-depth with ROI [11].

In order to investigate regional structural changes in the developing brain from late childhood to young adults, we spatially and temporally mapped brain maturation between the ages of 6–26 years using VBM and ROI-based method of high-resolution structural MRI. Each subject's graph was constructed by parcellation of the whole gray matter into 90 distinct regions, which contained both cortical lobes and subcortical regions, using the automated anatomical labeling (AAL) atlas. Moreover, total brain GM, WM, and CSF volumes were also assessed for linear age effects using multiple regressions.

## 2. Methods

### 2.1. Subjects

Brain imaging data were collected from 91 healthy ethnic Chinese (6–26 years old), including 33 late childhood (6-7 years, mean age 7.4 years, 14 female, 19 male), 33 early adolescence (9–11 years, mean age 10.3 years, 13 female, 20 male), and 25 young adults (18–26 years, mean age 22.7 years, 13 female, 12 male). Informed consent was obtained from the subjects and/or their parent as appropriate, and the study was approved by the Institutional Review Board of Beijing MRI Center for Brain Research.

### 2.2. MRI Acquisition

MRI was performed at the Beijing MRI Center for Brain Research of the Chinese Academy of Sciences using a 3 Tesla imager (Siemens, Erlangen, Germany) with a standard head coil. Three-dimensional, high-resolution anatomical scans were acquired by using an MPRAGE sequence with the following parameters: echo time = 3.01 ms; repetition time = 2,300 ms; flip angle = 9°; 176 coronal T1-weighted slices with a field of view = 256 × 240 mm and voxel sizes = 1 × 1 × 1 mm.

### 2.3. VBM Analysis

VBM analysis was performed using the DARTEL in SPM8 [8, 12, 13]. The processing steps are briefly explained below as follows: (1) estimate and write: the images were bias-corrected and segmented into GM, WM, and CSF; (2) DARTEL create template: a customized template was created for our study; (3) DARTEL existing template: once the study-specific template was created from the above step, the remaining subjects were registered nonlinearly to this template using DARTEL existing template module; (4) normalize to MNI space; (5) smooth: after normalizing and registering all subjects to MNI space, the resulting images were modulated (without including affine component) and smoothed using a full-width half-maximum (FWHM) of 8 mm; and (6) parametric statistics: the smoothed images were used for statistical inference.

For the statistical analysis, regional differences in gray matter voxel were tested by two-sample Student's *t*-test. To avoid possible edge effects between different tissue types, we excluded all voxels with gray matter values of 0.2 (absolute threshold masking). We applied a threshold of *P* < 0.05 FWE with an extent of 50 voxels across the whole brain.

To examine the relationship between variability in brain structure and age, the AAL atlas was used to define anatomic regions and the voxel of each region was reported. The gray matter volume of each ROI was extracted with Marsbar in SPM. The multiple regression was done based on AAL ROI, and the correlation (Pearson's* r*) between age and GMV at each region was calculated, and a significant threshold of *P* = 0.05 was used to illustrate local changes in GMV at each region.

## 3. Results

For all statistical parametric analyses, an extent threshold of 50 contiguous voxels was used and the results were corrected for multiple comparisons using the FWE approach (*P* < 0.05) at cluster level.

**Figure 1:**
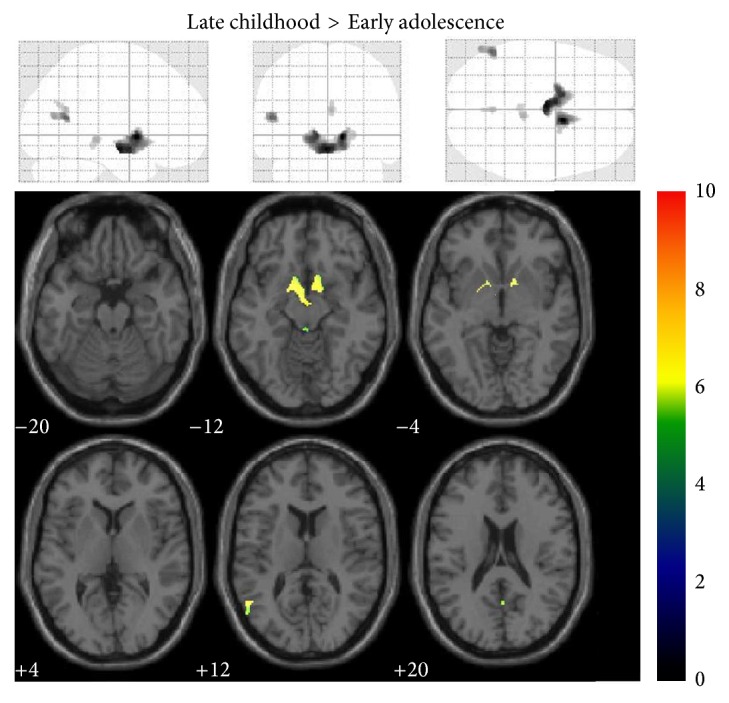
Late childhood minus early adolescence statistical parametric mapping. Traditionally presented* z*-score map (height threshold, 0.05; extent threshold, 50) for the gray matter voxel reductions observed between late childhood and early adolescence (upper). Voxels showing significant changes in gray matter mapped onto a normalized structural image in the axial plane (lower).

**Figure 2:**
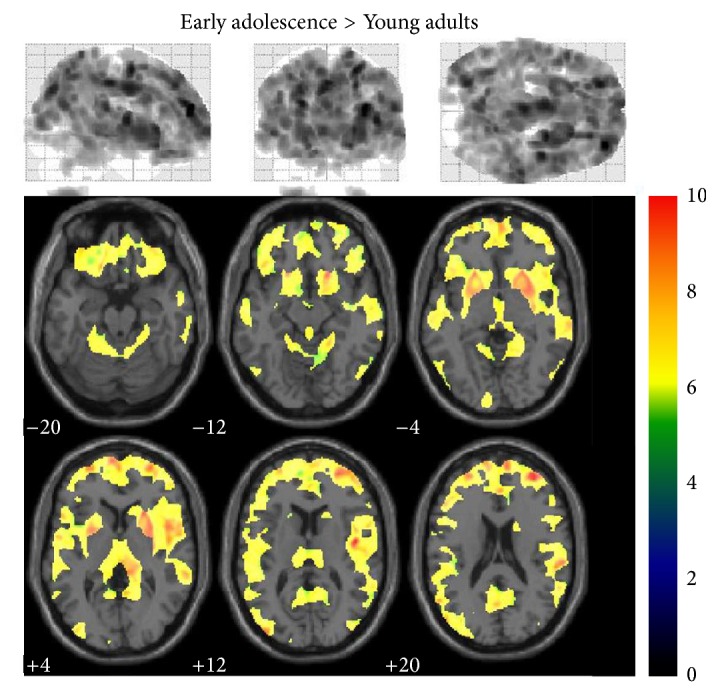
Early adolescence minus young adults statistical parametric mapping. Traditionally presented* z*-score map (height threshold, 0.05; extent threshold, 50) for the gray matter voxel reductions observed between early adolescence minus young adults (upper). Voxels showing significant changes in gray matter mapped onto a normalized structural image in the axial plane (lower).

Differences in GMV among late childhood, early adolescence, and young adults were analyzed. Localized differences in gray matter among the three groups were displayed as statistical parametric maps in Figures 1 and 2. The statistical parametric map in Figure 1 represented the loss of GMV between late childhood and early adolescence (e.g., late childhood minus early adolescence). Loss of GMV in early adolescence occurred in left olfactory, right precuneus, left and right caudates, left putamen, left and right pallidums, and left middle temporal gyrus, which showed statistical significance in comparison with late childhood (*P* < 0.05). Surprisingly, comparison between early adolescence and young adults showed an extensive GMV loss throughout the whole brain, and only three areas showed no significance, which were left amygdale, right amygdale, and left middle temporal pole (Figure 2, *P* > 0.05). However, when the same height and extent thresholds were used to assess gray matter increase between late childhood and early adolescence, as well as between early adolescence and young adults, no significant voxel was observed.

**Figure 3:**
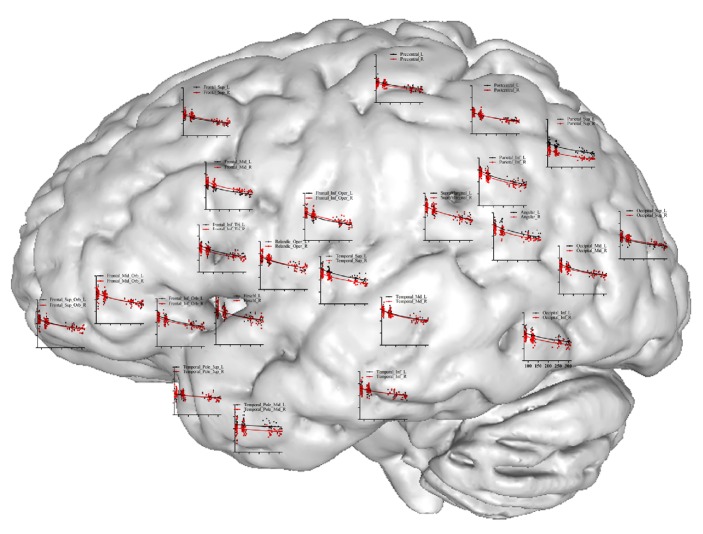
Scatterplot map of cortical lobes. Shown is a surface rendering of a human brain with scatterplots for GMV at various regions over the cortical lobes where the measurements were taken. Age in months is represented on the* x*-axis (range 50 to 300) and GMV on the* y*-axis (range 0.2 to 1.0). Red lines represent left hemisphere and black lines represent right hemisphere.

**Figure 4:**
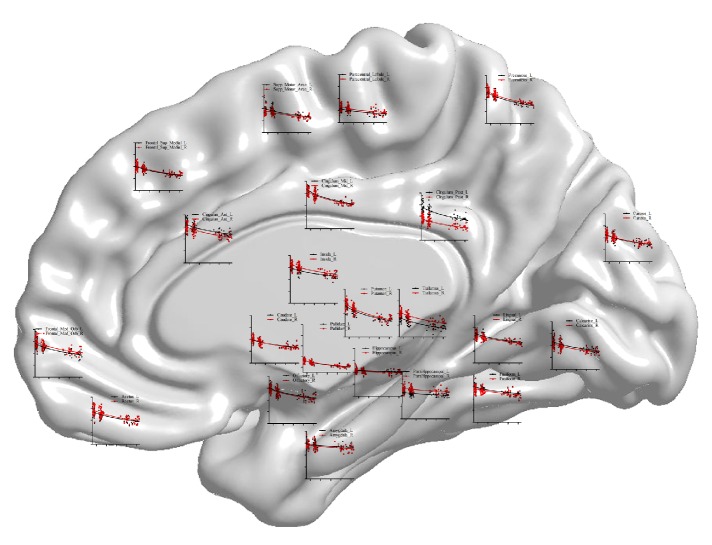
Scatterplot map of the subcortical regions. Shown is a surface rendering of a human brain with scatterplots for GMV at various anatomical points over the subcortical regions where the measurements were taken. Age in months is represented on the* x*-axis (range 50 to 300) and GMV on the* y*-axis (range 0.2 to 1.0). Red lines represent left hemisphere and black lines represent right hemisphere.

We constructed statistical maps of linear age effects on GMV for all 91 subjects. Multiple regression was used to determine the variance in GMV predicted by the age variable. Multiple regression analyses revealed significant, linear age effects over most areas on both cortical lobes and subcortical regions (Figures 3-4). Scatterplots of these effects showed a dramatic decline in GMV from the ages 6 to 26 years. Statistical analysis showed that among the 90 areas only the left middle temporal pole shows no correlation with age (Figure 3, regression coefficient *β* = −0.194, *P* = 0.066). Furthermore, we divided the cortical lobes into frontal, parietal, occipital, insular, and temporal lobes and compared the loss ratio of GMV [note: loss ratio of GMV = (average value of GMV in late childhood − average value of GMV in young adults)/average value of GMV in late childhood]. Intriguingly, the most loss ratio of GMV in these lobes appeared in parietal (25.12%, *P* < 0.05 when compared with the other four lobes), then in frontal (21.51%) and occipital lobes (19.59%) (*P* = 0.146, when compared between the two lobes), and the least loss ratio appeared in insular (15.98%) and temporal lobes (15.38%) (*P* = 0.648 when compared between the two lobes and *P* < 0.05 when compared with the other three lobes). In addition, maps of the subcortical regions also showed pronounced GMV reduction (Figure 4). Notably, the sharp decline was observed in basal ganglia and thalamus, in which a 33% decline in GMV was observed in global pallidus especially. In contrast, the less loss of GMV occurred in hippocampus, only 0.05%.

**Figure 5:**
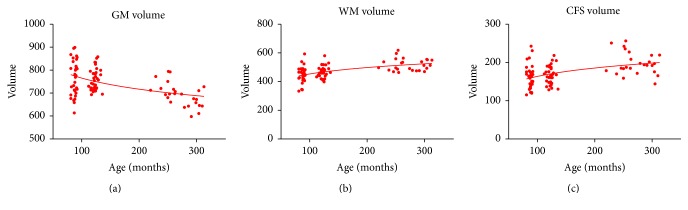
Volume graphs. Scatterplots of the linear effects of age on total brain gray matter (a), white matter (b), and CFS (c) volume.

Total brain GM, WM, and CSF volumes were also assessed for linear age effects using multiple regression. The effect of age on GM volume in this sample was highly significant (*β* = −0.501, *P* < 0.001, Figure 5(a)). The decrease in GM volume lasted consistently from late childhood to young adults. However, a strong linear increase in both WM volume (*β* = 0.521, *P* < 0.001, Figure 5(b)) and CSF volume (*β* = 0.457, *P* < 0.001, Figure 5(c)) was observed with increasing age.

## 4. Discussion

Results from this study showed regionally specific brain growth between 6 and 26 years old by testing for age effects on a voxel by voxel basis using parametric statistics. Statistical maps of local gray matter changes revealed the earliest decline occurring in basal ganglia, left olfactory, right precuneus, and left middle temporal gyrus. In addition, the trajectory of maturational and aging showed a linear decline in GMV on both lobes and subcortical regions. More specifically, the most loss of gray matter was observed in the parietal lobe and basal ganglia, whereas the less loss occurred in the temporal lobe and hippocampus, especially in the left middle temporal pole, which showed no decline until 26 years old.

Previous studies have revealed a shifting pattern of gray matter loss [1, 4, 5]. Studies evaluating the order in which areas of the brain mature have found that the first to mature are those that perform the most basic functions (e.g., sensory input and movement), followed by areas involved in spatial orientation and language and then more advanced functions that integrate input from the senses, reasoning, and additional executive functions. Importantly, this suggests that there is an indirect relationship between evolutionary age and the age of the human when areas of the brain mature; that is, the evolutionarily younger higher order associated cortices mature later in life than the evolutionarily younger cortical areas with the former integrating information from the latter. In this study, the pattern of the decline in gray matter volume matched the phenotype observed previous studies, where the fastest and less occurred in the parietal and temporal lobes, respectively. We hypothesize that this decrease in gray matter is correlated with an increase in peripheral neuropil myelination, because brain growth would halt if the notable cortical thinning was from the regressive changes alone. Along these lines, decreases in MRI are correlated with a loss in macromolecules, particularly myelin. Chronologically, myelination occurs in the motor and sensory systems prior to the temporal and frontal lobes [14, 15], which is consistent with our observations. In this study, the volume of the white matter is in the process of measuring gray matter [5, 16]. It was found the volume of white matter rises with age, further supporting that the loss of gray matter is related to the increase in cerebral myelination.

When assessing the loss of basal ganglia, it was found it first and most severely occurs in the gray matter, most likely due to a combination of deposition of iron, myelination, and regression [17]. Characterization of the density of gray matter in the brains of healthy 12–16- and 23–30-year-olds found subcortical region loss primarily occurred in the putamen and globus pallidus of the striatum [18], which is consistent with our results. The basal ganglia is involved in motor and cognitive functions, for example, learning, which are likely fully developed by late childhood. However, data presented in both our study and previously published studies have revealed a decline in the basal ganglia at an early age. When considering which factors are involved in the decrease in gray matter in addition to myelination, [2, 19] iron deposition should be a candidate. A study of 143 healthy women of up to 40 years of age used susceptibility weighted imaging and found a direct correlation between iron deposition in the globus pallidus and age. Also, one study noted iron deposition has trended an increase with age in the putamen in people of up to 50–70-year-olds, while another study showed iron deposition peaks at 60 years in the head of the caudate nucleus. [20]. Another study on 138 healthy people aged 19 to 75 years using whole-brain voxel-wise analysis noted a general age-related degeneration and a substantial increase in iron levels in the basal ganglia [14]. These changes in iron content reflect a complex process and are difficult to interpret. Normally, healthy oligodendrocytes in the human brain strongly stain for ferritin, iron, and transferrin [21]. Therefore, age-related differences in the oligodendroglia may account for some of the observed age-related differences in MRI as well as the levels of myelination due to the critical role oligodendrocytes play in the myelin status. It is currently unknown to what degree the reductions in gray matter are driven by iron deposition and/or myelination. Unfortunately, the mechanism behind the slower rate of loss in the gray matter of the hippocampus is more difficult to explain than changes in the basal ganglia.

Previously, age-dependent changes in the brain have been suggested to follow a nonlinear trajectory [22, 23]. This present study included participants aged 6 to 26 years, but no 12- to 18-year-olds, likely increasing the sensitivity of our analysis to linear versus higher order differences. This work found a reduction in gray matter that supports an increasing rate of decline with advancing age. In addition, both genetics and environmental factors may influence the developmental trajectories in the brain in addition to age [24]. Previous studies on twins have shown that genetic factors drive a number of individual differences in human anatomy [25]. A follow-up study on 326 twins and 158 singletons determined that the majority of the variation observed in the corpus callosum can be explained by additive genetic effects. In addition, a relatively small, but significant, effect from environmental factors was noted in multiple neuroanatomic regions, especially in the thalamus, lateral ventricles, and basal ganglia [26]. Another study compared brain development between Chinese and American children and adolescents and found significant differences in the intensity of both gray and white matter, as well as the cerebral and gray matter volume and a number of critical structures [27]. These complex effects emphasize the difficulty of drawing conclusions about brain development based on studies with small cohorts. Therefore, both longitudinal and cross-sectional studies are required to truly understand the effect of aging on developmental trajectory, which are possible using MRI, but not invasive histology approaches.

## 5. Conclusion

Our data provide new insights into the spatial and temporal patterns of age-related change in brain structure from 6 to 26 years old, not only globally between GM, WM, and CSF compartments, but also locally within regions of the GM. The result of large numbers of subjects in this study contributes to the reliable data for further studying of the age-related brain development but also highlights the reliability of VBM as a tool for detecting subtle structural brain changes in normal subjects.

## Abbreviations

VBM: Voxel-based-morphometry
ROI: Region-of-interest
MRI: Magnetic resonance imaging
GMV: Gray matter volume
GM: Gray matter
WM: White matter
CSF: Cerebrospinal fluid.

## References

[1] Giedd J. N., Blumenthal J., Jeffries N. O. (1999). Brain development during childhood and adolescence: A Longitudinal MRI Study. *Nature Neuroscience*.

[2] Sowell E. R., Thompson P. M., Tessner K. D., Toga A. W. (2001). Mapping continued brain growth and gray matter density reduction in dorsal frontal cortex: inverse relationships during postadolescent brain maturation. *Journal of Neuroscience*.

[3] Gogtay N., Giedd J. N., Lusk L. (2004). Dynamic mapping of human cortical development during childhood through early adulthood. *Proceedings of the National Academy of Sciences of the United States of America*.

[4] Toga A. W., Thompson P. M., Sowell E. R. (2006). Mapping brain maturation. *Trends in Neurosciences*.

[5] Sowell E. R., Peterson B. S., Thompson P. M., Welcome S. E., Henkenius A. L., Toga A. W. (2003). Mapping cortical change across the human life span. *Nature Neuroscience*.

[6] Paus T., Zijdenbos A., Worsley K. (1999). Structural maturation of neural pathways in children and adolescents: In Vivo Study. *Science*.

[7] Ashburner J., Friston K. J. (2000). Voxel-based morphometry—the methods. *NeuroImage*.

[8] Good C. D., Johnsrude I. S., Ashburner J., Henson R. N. A., Friston K. J., Frackowiak R. S. J. (2001). A voxel-based morphometric study of ageing in 465 normal adult human brains. *NeuroImage*.

[9] Tisserand D. J., Pruessner J. C., Sanz Arigita E. J. (2002). Regional frontal cortical volumes decrease differentially in aging: an MRI study to compare volumetric approaches and voxel-based morphometry. *NeuroImage*.

[10] May A., Ashburner J., Büchel C. (1999). Correlation between structural and functional changes in brain in an idiopathic headache syndrome. *Nature Medicine*.

[11] Testa C., Caroli A., Roberto V., Frisoni G. B. (2006). Structural brain imaging in patients with cognitive impairment in the year 2015. *Future Neurology*.

[12] White N. S., Alkire M. T., Haier R. J. (2003). A voxel-based morphometric study of nondemented adults with Down Syndrome. *NeuroImage*.

[13] Rajagopalan V., Yue G. H., Pioro E. P. (2014). Do preprocessing algorithms and statistical models influence voxel-based morphometry (VBM) results in amyotrophic lateral sclerosis patients? A systematic comparison of popular VBM analytical methods. *Journal of Magnetic Resonance Imaging*.

[14] Callaghan M. F., Freund P., Draganski B. (2014). Widespread age-related differences in the human brain microstructure revealed by quantitative magnetic resonance imaging. *Neurobiology of Aging*.

[15] Kemper T. L. (1994). Neuroanatomical and neuropathological changes during aging and dementia. *Clinical Neurology of Aging*.

[16] Bartzokis G., Beckson M., Lu P. H., Nuechterlein K. H., Edwards N., Mintz J. (2001). Age-related changes in frontal and temporal lobe volumes in men: a magnetic resonance imaging study. *Archives of General Psychiatry*.

[17] Gregory A., Hayflick S. (2013). *Neurodegeneration with Brain Iron Accumulation Disorders Overview*.

[18] Sowell E. R., Thompson P. M., Holmes C. J., Jernigan T. L., Toga A. W. (1999). In vivo evidence for post-adolescent brain maturation in frontal and striatal regions. *Nature Neuroscience*.

[19] Barkovich A. J., Kjos B. O., Jackson D. E., Norman D. (1988). Normal maturation of the neonatal and infant brain: MR imaging at 1.5 T. *Radiology*.

[20] Wang D., Li Y.-Y., Luo J.-H., Li Y.-H. (2014). Age-related iron deposition in the basal ganglia of controls and Alzheimer disease patients quantified using susceptibility weighted imaging. *Archives of Gerontology and Geriatrics*.

[21] Morris C. M., Candy J. M., Oakley A. E., Bloxham C. A., Edwardson J. A. (1992). Histochemical distribution of non-haem iron in the human brain. *Acta Anatomica*.

[22] Blakemore S.-J. (2012). Imaging brain development: the adolescent brain. *NeuroImage*.

[23] Shaw P., Kabani N. J., Lerch J. P. (2008). Neurodevelopmental trajectories of the human cerebral cortex. *Journal of Neuroscience*.

[24] Giedd J. N., Rapoport J. L. (2010). Structural MRI of pediatric brain development: what have we learned and where are we going?. *Neuron*.

[25] Wallace G. L., Eric Schmitt J., Lenroot R. (2006). A pediatric twin study of brain morphometry. *Journal of Child Psychology and Psychiatry*.

[26] Schmitt J. E., Wallace G. L., Rosenthal M. A. (2007). A multivariate analysis of neuroanatomic relationships in a genetically informative pediatric sample. *NeuroImage*.

[27] Xie W., Richards J. E., Lei D., Lee K., Gong Q. (2015). Comparison of the brain development trajectory between Chinese and U.S. children and adolescents. *Frontiers in Systems Neuroscience*.

